# Flexural Strength Prediction of Steel Fiber-Reinforced Concrete Using Artificial Intelligence

**DOI:** 10.3390/ma15155194

**Published:** 2022-07-27

**Authors:** Dong Zheng, Rongxing Wu, Muhammad Sufian, Nabil Ben Kahla, Miniar Atig, Ahmed Farouk Deifalla, Oussama Accouche, Marc Azab

**Affiliations:** 1 School of Architectural Engineering, Ningbo Polytechnic, Ningbo 315800, China; wurongxing98@163.com; 2 School of Civil Engineering, Southeast University, Nanjing 210096, China; 3 Department of Civil Engineering, College of Engineering, King Khalid University, Abha 61421, Saudi Arabia; nbohlal@kku.edu.sa; 4 Laboratory of Systems and Applied Mechanics, Tunisia Polytechnic School, University of Carthage, La Marsa, Tunis 2078, Tunisia; miniar.atig@gmail.com; 5 Department of Civil Engineering, The Higher National Engineering School of Tunis, University of Tunis, Tunis, Tunisia; 6 Structural Engineering and Construction Management Department, Faculty of Engineering and Technology, Future University in Egypt, Cairo 11835, Egypt; 7 College of Engineering and Technology, American University of the Middle East, Egaila 54200, Kuwait; oussama.accouche@aum.edu.kw (O.A.); marc.azab@aum.edu.kw (M.A.)

**Keywords:** concrete, steel fiber, steel fiber-reinforced concrete, flexural strength, mechanical characteristics, construction materials

## Abstract

Research has focused on creating new methodologies such as supervised machine learning algorithms that can easily calculate the mechanical properties of fiber-reinforced concrete. This research aims to forecast the flexural strength (FS) of steel fiber-reinforced concrete (SFRC) using computational approaches essential for quick and cost-effective analysis. For this purpose, the SFRC flexural data were collected from literature reviews to create a database. Three ensembled models, i.e., Gradient Boosting (GB), Random Forest (RF), and Extreme Gradient Boosting (XGB) of machine learning techniques, were considered to predict the 28-day flexural strength of steel fiber-reinforced concrete. The efficiency of each method was assessed using the coefficient of determination (R^2^), statistical evaluation, and k-fold cross-validation. A sensitivity approach was also used to analyze the impact of factors on predicting results. The analysis showed that the GB and RF models performed well, and the XGB approach was in the acceptable range. Gradient Boosting showed the highest precision with an R^2^ of 0.96, compared to Random Forest (RF) and Extreme Gradient Boosting (XGB), which had R^2^ values of 0.94 and 0.86, respectively. Moreover, statistical and k-fold cross-validation studies confirmed that Gradient Boosting was the best performer, followed by Random Forest (RF), based on reduced error levels. The Extreme Gradient Boosting model performance was satisfactory. These ensemble machine learning algorithms can benefit the construction sector by providing fast and better analysis of material properties, especially for fiber-reinforced concrete.

## 1. Introduction

The incorporation of steel fibers in concrete improves the mechanical characteristics, i.e., compressive strength, flexural strength, and tensile strength, making the concrete more tough and resistible to cracks, as reported in previous works of literature [1,2,3,4,5,6]. Steel fiber-reinforced concrete significantly increased flexural strength compared to regular concrete [7]. The flexural behavior of SFRC beams was investigated and it was found that increasing the steel fiber content improved strength, toughness, and load-bearing capability [8]. The addition of up to 15% steel fibers to concrete increased the frost resistance and longevity of the concrete [9]. The FS of SFRC was investigated concerning curing time and fiber volume fraction. It was discovered that high-performance steel fiber and a high fiber volume fraction are necessary for SFRC flexural toughness [10]. The influence of fiber content and concrete strength on SFRC flexural behavior was investigated experimentally [11]. The addition of silica fume and steel fiber content to high-strength SFRC improved its toughness [12]. Analytical and experimental results were given on the flexural response of SFRC beams. According to the findings, the increased steel fiber volume enhanced flexural strength, deflection capacity, and post-peak ductility [13]. The mechanical characteristics of high-strength concrete were studied concerning steel fiber content and coarse particle size. The results showed that increasing the fiber content substantially increased the SFRC’s compressive and flexural toughness [14]. Natural and synthetic fibers have been utilized to improve the mechanical properties of concrete and cementitious materials [15,16,17,18,19,20,21,22,23,24,25].

Machine learning (ML) techniques have recently been established, which play a vital role in the civil engineering industry by predicting the mechanical characteristics of concrete with a high degree of precision. ML is a branch of computer science that automates the creation of analytical models and is used to analyze data. ML algorithms are designed to learn from previously collected data. ML has gained popularity due to its ability to handle vast amounts and types of data. In addition, the computational procedure is less costly and more effective. As a consequence, models for evaluating massive and complicated data, as well as for delivering faster and more accurate results, may be created quickly and automatically. The application of these models results in highly exact predictions, allowing for more competent judgments and intelligent actions to be made in real-time without the need for human intervention [26]. The development of ML models to predict concrete strength is now underway to decrease the wastage of materials and experimental cycles. Artificial intelligence (AI) techniques like machine learning (ML) are among the most advanced modeling methodologies used in civil engineering. These approaches use input variables to model responses. Using supervised machine learning methodologies, researchers have recently focused on the compressive strength of concrete and other strength qualities such as flexural strength, tensile strength, and concrete durability. The M5P model was used by Behnood et al. to investigate concrete’s compressive, flexural, and split tensile strength [27]. Several studies have attempted to predict concrete strength characteristics [28,29,30,31,32,33,34,35]. Machine learning methods are employed to forecast concrete strength [36,37,38,39,40,41,42,43,44,45,46,47,48,49] and the durability of concrete [50,51,52]. Bagging regression (BR) and gradient boosting (GB) models based on a variation of the bootstrap aggregation decision tree (DT) method have been shown in several studies to outperform other stand-alone ML models in terms of concrete strength prediction accuracy [53,54,55,56].

The use of machine learning to assess the strength properties of SFRC is novel. However, it is challenging due to the additional factors compared to conventional concrete, such as fiber type, fiber length, fiber diameter, and fiber content. The development of reliable algorithms to forecast the mechanical characteristics of fiber-reinforced concrete is currently ongoing. This study aims to see how three ensembled machine learning algorithms may be utilized to predict the flexural strength of SFRC. Gradient Boosting (GB), Random Forest (RF), and Extreme Gradient Boosting (XGB) are three machine learning (ML) algorithms developed and compared in this work to predict the flexural strength of SFRC. Correlation coefficients (R^2^) and statistical tests were used to assess the effectiveness of each strategy. In addition, the validity of each approach was validated using k-fold assessment and error distributions. This study is notable since it employs ensemble ML algorithms to predict the FS of SFRC and minimize the experimentation process because experimental works need a significant amount of human work, experimentation costs, and time for material gathering, casting, curing, and testing. Since several factors impact the flexural strength of SFRC, including cement, water, aggregate, additives, fiber volume, fiber length, and fiber diameter, determining their total effect is challenging. With minimum effort, machine learning approaches can determine the combined impact of its components. Since various investigations have been undertaken to estimate the FS of SFRC, ML algorithms require a data set, which may be acquired from prior research. The information gathered may subsequently be used to train machine learning algorithms and predict material strength.

## 2. Data Description

The dataset was created exclusively utilizing data from hook-end steel fibers concrete. The data were acquired from 17 sources [8,11,12,13,14,57,58,59,60,61,62,63,64,65,66,67,68]. The factors that significantly impacted the outcome were selected and processed. Consequently, the dataset comprises ten distinct elements, including input and output data. These 10 components were taken into account while predicting SFRC flexural strength, and each of these variables influences SFRC flexural strength.

### 2.1. Water and Cement

According to prior research, the water-to-cement ratio significantly influences concrete strength. Abbass et al. reported that when the water–cement ratio increases, the flexural strength and compressive strength decrease [68]. Reddy et al. discovered that the water–cement ratio considerably influenced the flexural and compressive strength of self-consolidating concrete [69]. In a scientific investigation, Nili et al. demonstrated that SFRC obtained better flexural strength with a lower water–cement ratio [70]. Merve AÇIKGENÇ et al. investigated the relationship between SFRC splitting tensile and flexural strength using various cement dosages and water–cement ratios [71]. Wei Li investigated the impact of the water–cement ratio on concrete performance and discovered that when the water–cement ratio rises, the concrete strength diminishes [72]. M. S. Ahmad Shah et al. experimentally examined the flexural strength of concrete with various water–cement ratios and concluded that the flexural strength increased as the water–cement proportion increased [73]. Chang Joon Lee et al. looked at how the water–cement ratio and fiber content affected the flexural toughness of SFRC. A lower water–cement ratio and higher fiber volume result in a faster flexural toughness convergence rate [74]. E. K. Z. Balanji investigated how different water–cement ratios and steel fiber content affected the mechanical characteristics and impact resistance of steel fiber concrete. The influence of steel fibers on mechanical factors and impact resistance was more beneficial when the water–cement ratio was lower [75]. The water–cement ratio is chosen as a variable impacting the flexural strength of concrete in light of the cited literature.

### 2.2. Sand and Aggregate

The influence of sand and aggregate proportion on the strength qualities of SFRC has been recognized as a key factor. Kim et al. found that the higher proportion of sand to aggregate boosted the compressive and flexural strength of SFRC [76]. The amounts of sand and aggregate in concrete caused a noticeable difference in flexural and compressive strength, according to Chitlange et al. [77]. K. B. Dashrath et al. provided a comparative investigation of the flexural strength of concrete with varied aggregate quantities and kinds [78]. El-Ariss studied the impact of the water–cement ratio, sand, and gravel, and their various proportions and curing process on the concrete strength [79]. U. M. Tarek et al. analyzed the effects of various sand–aggregate ratios on concrete strength properties and determined the ideal sand–aggregate ratio [80]. M. Sunarso and colleagues studied the impacts of sand–aggregate fraction and additive dose on numerous characteristics of high-strength concrete [81]. The sand to aggregate ratio was considered as a key feature in the ML models design due to its importance in the strength qualities of concrete.

### 2.3. Superplasticizer

A superplasticizer is a water-reducing chemical used in the manufacture of concrete to increase its strength qualities. To improve the mechanical properties of concrete, superplasticizer and pozzolanic additives were utilized by M. Khan and M. Ali [82]. According to Aruntas et al., increased superplasticizer concentration enhanced concrete slump and strength properties [83]. Consequently, a superplasticizer was included in the ML models to assess its impact on SFRC flexural strength.

### 2.4. Silica Fume

To improve the strength qualities of concrete, silica fume has been utilized in varying quantities. Köksal et al. reported that the compressive and flexural strength of concrete improved with the increased silica fume [12]. The concrete flexural strength was notably enhanced when steel fibers and silica fume were employed simultaneously, according to M. Nili and V. Afroughsabet [84]. M. Shafieyzadeh discovered that substituting up to 7.5% of the cement with silica fume increases the flexural strength of concrete by 15% [85]. M. Shmlls et al. found that the combined dosage of silica fume and fly ash enhanced the strength properties of concrete [86]. The incorporation of silica fume was identified as a component that influences the strength qualities of SFRC.

### 2.5. Fly Ash

Fly ash increases the workability of plastic concrete as well as the strength properties of hardened concrete. R. M. K Saravana and A. Sumathi discovered that the addition of fly ash into SFRC enhanced the concrete strength over time [87]. M. A. Challoob et al. investigated the effect of fly ash and steel fibers on the strength of pozzolana cement concrete [88]. A.K. Saha found that the concrete strength increased gradually when the fly ash was introduced [89]. P. Nath and P. Sarker stated that the durability characteristics of high-strength concrete improved with the partially addition of fly ash [90]. Thus, fly ash was picked as a variable due to its relevance to concrete qualities.

### 2.6. Steel Fiber Volume, Length and Diameter

The steel fiber proportion, length, and thickness have a notable impact on the flexural strength of concrete, as reported in the literature. Yazici et al. found that the compressive and flexural strength of concrete improved with increased steel fiber [7]. Köksal et al. revealed that SFRC compressive and flexural strength improved due to an experimental investigation utilizing fiber volume fractions up to 1% [12]. A. A. Jhatial et al. determined that the increased content of steel fibers improved the flexural and compressive strength [91]. H. K. Hussain et al. revealed that steel fibers in concrete remarkably enhanced the strength and durability properties of hardened concrete. The flexural strength significantly increased with the incorporation of hooked end textured steel fibers [92]. According to Hyun-Oh Shin et al., in terms of flexural behavior of ultra-high-performance fiber-reinforced concrete under uniaxial and biaxial stress states, the straight steel fiber is the most effective [93]. As a result, ML models must include fiber volume, length, and diameter as variables.

**Figure 1:**
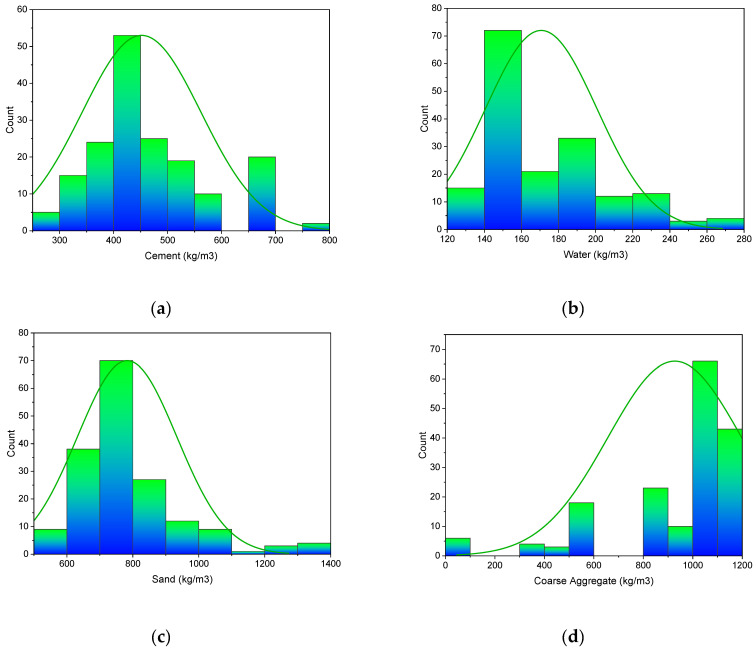

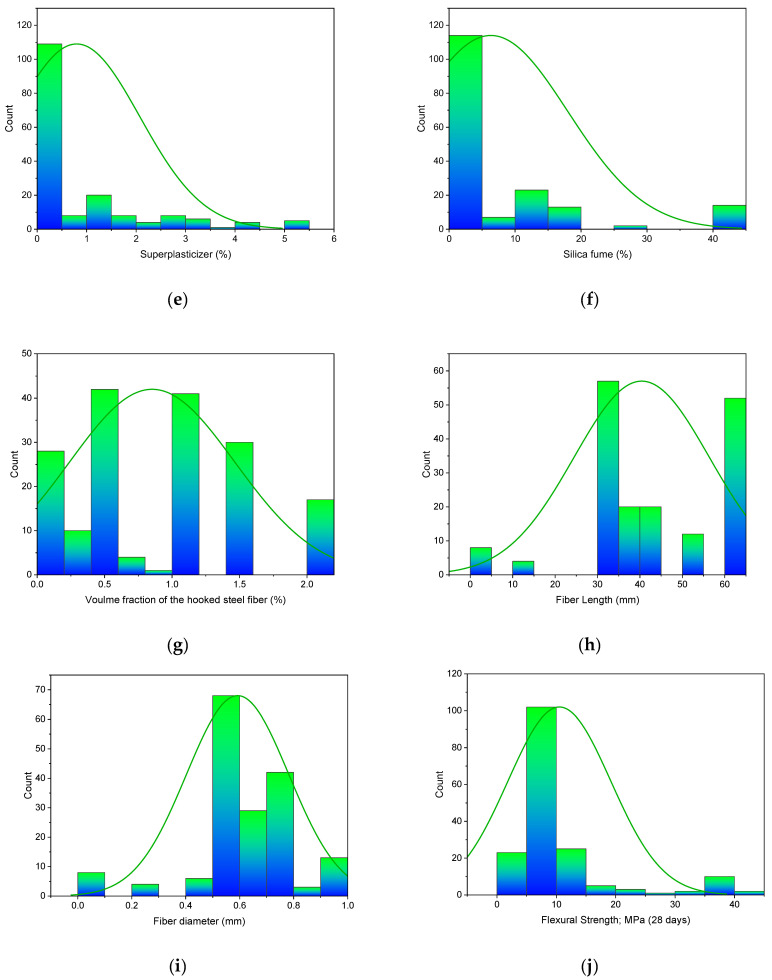
Input variable; relative frequency pattern: (**a**) Cement; (**b**) Water; (**c**) Sand; (**d**) Coarse aggregate; (**e**) Super-plasticizer; (**f**) Silica fume; (**g**) Volume fraction of steel hooked fibers; (**h**) Fiber length; (**i**) Fiber diameter; (**j**) Flexural strength.

Machine learning techniques require a variety of input parameters to obtain the desired outcome. The data used to calculate the 28 days of SFRC’s flexural strength were gathered from the literature. Cement, water, sand, coarse aggregate content, superplasticizer, silica fume, fly ash, hooked steel fiber volume, fiber length, and fiber diameter were all included as inputs in the model, with just one variable–flexural strength–as an outcome. For the 28-day SFRC flexural strength prediction, this study employed 173 data points (mix proportions). The statistical analysis results of the input variables, such as mean, standard error, median, mode, standard deviation, range, minimum, and maximum values, are shown in Table 1. In addition, Figure 1 shows the relative frequency pattern distribution of all input parameters.

## 3. Research Strategy

**Figure 2:**
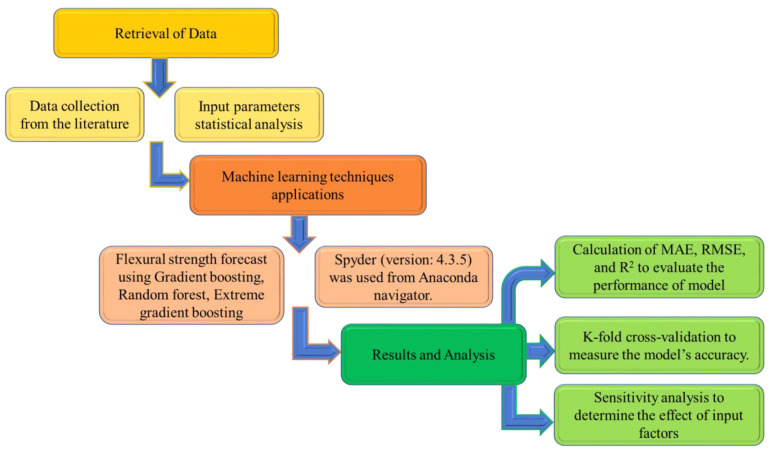
Methodology of research in order.

Anaconda software was used to build the machine learning models, employing python code. The Anaconda navigator is a graphical user interface included in the Anaconda software that allows programs to run that give direction via Conda packages, channels, and environments without requiring command-line skills. It also provides Python and R programming languages for data science and machine learning applications, focusing on package creation and maintenance. This work used three ensembled techniques to estimate the flexural strength of SFRC, i.e., Gradient Boosting (GB), Random Forest (RF), and Extreme Gradient Boosting (XGB). The Anaconda navigator’s Spyder (version: 4.3.5) was used for model execution. The R^2^ value of the projected outcome from all models was used to gauge the degree of accuracy. R^2^ values typically vary from 0 to 1, with a more significant number implying more accuracy in predicting the measured and projected results. Statistical checks, error evaluation (including MAE, RMSE), and k-fold cross-validation were conducted to examine the models’ performance. A sensitivity analysis was carried out to check the impact of all input factors. Figure 2 illustrates the research strategy.

## 4. Results and Discussions

### 4.1. Statistical Analysis Explanation

**Figure 3:**
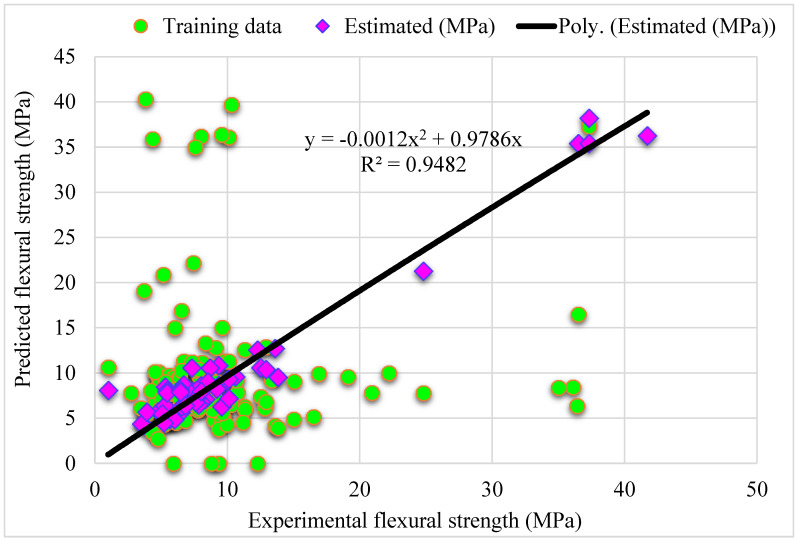
Relationship for R-F model: Experimental and estimated results.

**Figure 4:**
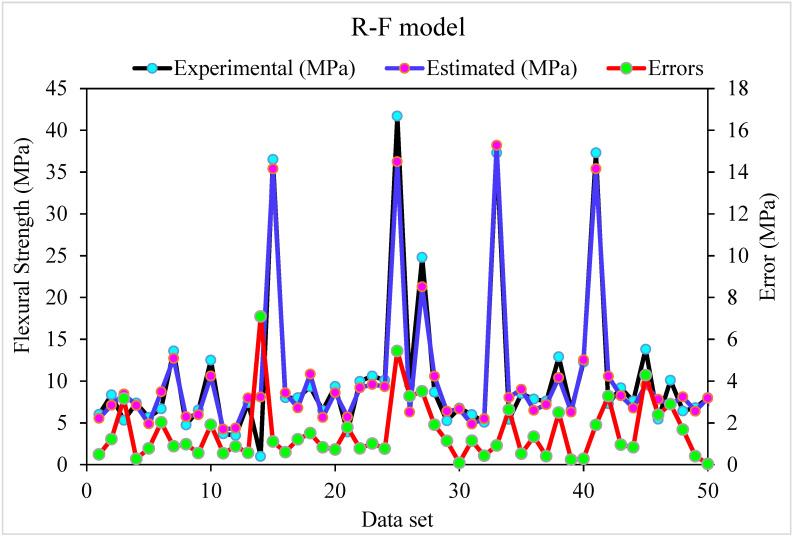
Experimental, estimated and, error values for R-F model.

Figure 3 shows a trend of statistical analysis using the R-F model to compare actual and anticipated SFRC flexural strength after 28 days. The R-F produces results within the allowed range and slight variations between predicted and actual outcomes. The R^2^ = 0.94 indicates that the model is effective at estimating outcomes. Figure 4 depicts the R-F model’s deviations and the distribution of investigational and projected outcomes. The distribution’s highest, lowest, and average error values were 7.09, 0.036, and 1.50 MPa, respectively. It was revealed that 52% of the incorrect readings were less than 1 MPa, 44% were between 1–5 MPa, and 3.8% were higher than 5 MPa. These statistics indicate the degree of agreement between expected and actual results.

**Figure 5:**
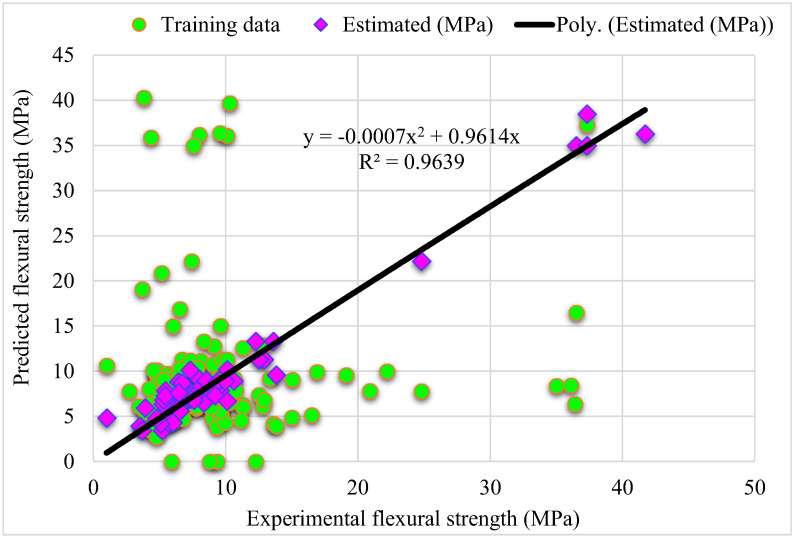
Experimental and estimated results connection for G-B model.

**Figure 6:**
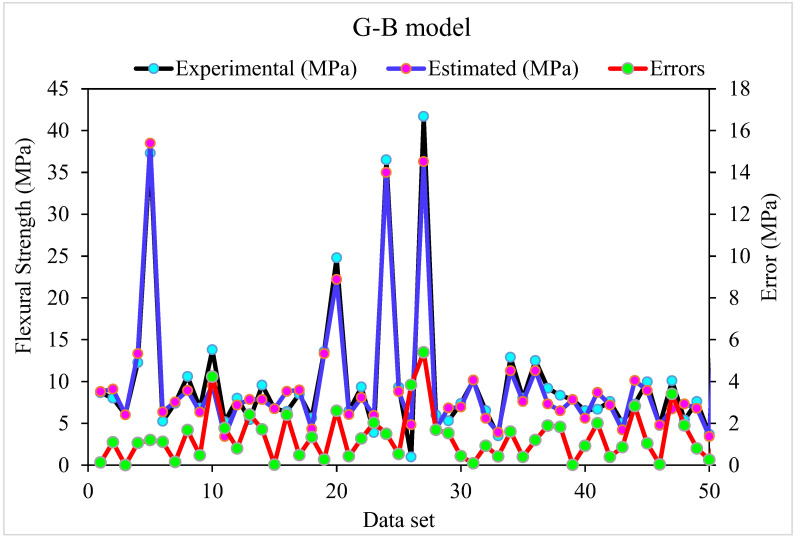
Experimental, estimated and, error distribution for G-B model.

The outputs of the G-B model are depicted in Figure 5 and Figure 6. Figure 5 shows the relationship between actual and expected outcomes, with R^2^ = 0.96–higher than the R-F model–showing that the G-B approach outperforms the R-F. The distribution of actual and predicted values–as well as errors–in the G-B model is depicted in Figure 6. The distribution’s maximum, minimum, and average error values were 5.4, 0.0026, and 1.34 MPa, respectively. According to the data, 42% of incorrect readings were less than 1 MPa, 56% were between 1 and 5 MPa, and 2% were greater than 5 MPa. Based on the R^2^ and error distribution of the R-F and G-B models, the G-B model can more accurately predict the SFRC flexural strength.

**Figure 7:**
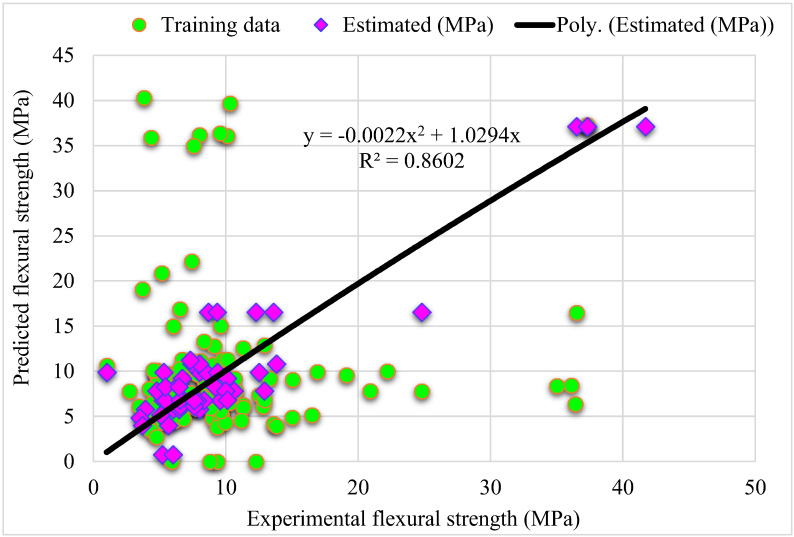
Experimental and estimated outcomes relationship for XGB model.

**Figure 8:**
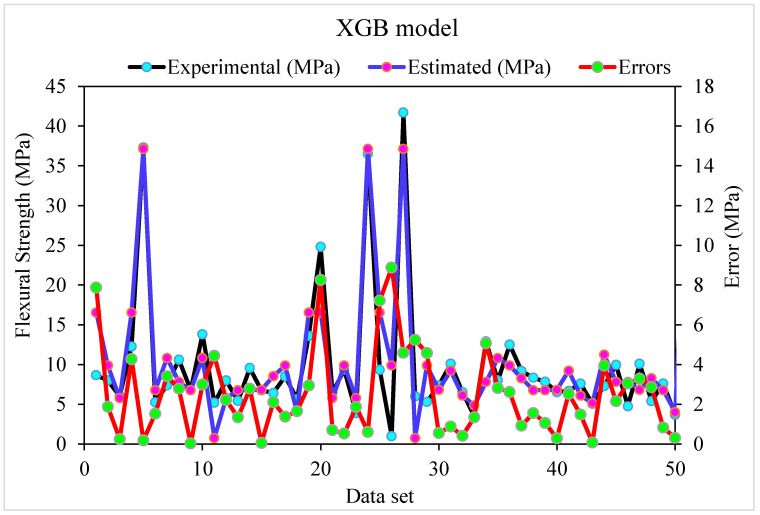
Experimental, estimated values and, error distribution for XGB model.

Figure 7 depicts the relationship between actual and predicted outcomes for the XGB model. The R^2^ value for the XGB model is 0.86, indicating that it is less precise than the R-F and G-B models. Figure 8 also illustrates the XGB model’s actual and anticipated values and errors distribution. The highest, lowest, and mean errors were 8.88, 0.036, and 2.43 MPa, respectively. According to the findings, 30% of the erroneous values were less than 1 MPa, 58% were between 1 MPa and 5 MPa, and 12% were higher than 5 MPa. Due to reduced inaccuracy and more excellent R^2^ readings, the G-B model was more precise than the R-F and XGB models in this study. Furthermore, ensembled ML approaches such as R-F, G-B, and XGB employed sub-models to obtain the best evaluation, resulting in flawless results. Consequently, ML techniques, i.e., G-B and R-F, were shown to be more accurate than XGB in predicting outcomes in this investigation.

### 4.2. Cross-Validation Using K Fold

The k-fold cross-validation approach is used to verify the model’s validity during execution. This method is frequently used to verify the accuracy of a model in which the data set is spread out and divided into ten groups [94,95,96]. The model was tested with one group, while the other nine were used for training. Overall, 70% of the data set was used in the model training process, with the remaining 30% used to evaluate the models. This process requires randomly dividing the set of observations into k groups or folds of roughly similar size. The first fold is used as a validation set, while the following k-1 folds are used to fit the procedure. The model is deemed more accurate if the R^2^ value is high and the errors, such as MAE and RMSE, are low. The process needs to be repeated 10 times to provide a satisfactory result. This comprehensive technique is essential for the model’s excellent accuracy. Furthermore, as indicated in Table 2, all models were statistically analyzed as errors (MSE and RMSE). Statistical analysis was used to assess the models’ reaction to estimation, using Equations (1) and (2) from the literature [97].

(1) $$\mathrm{MAE}=\frac{1}{n}\overset{n}{\underset{i=1}{\sum }}\left|x_{i}-x\right|$$

(2) $$\mathrm{RMSE}=\sqrt{\sum \frac{{\left(y_{pred}-y_{ref}\right)}^{2}}{n}}$$

where n = total number of sampled data. x, $y_{ref}$ = reference values of data sample. $x_{i}$, $y_{pred}$ = model-predicted values.

**Figure 9:**
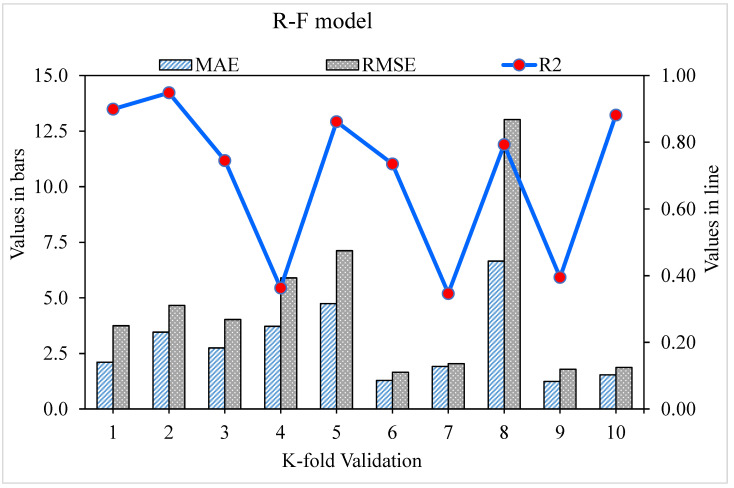
Random Forest model with K-fold cross-validation representation.

**Figure 10:**
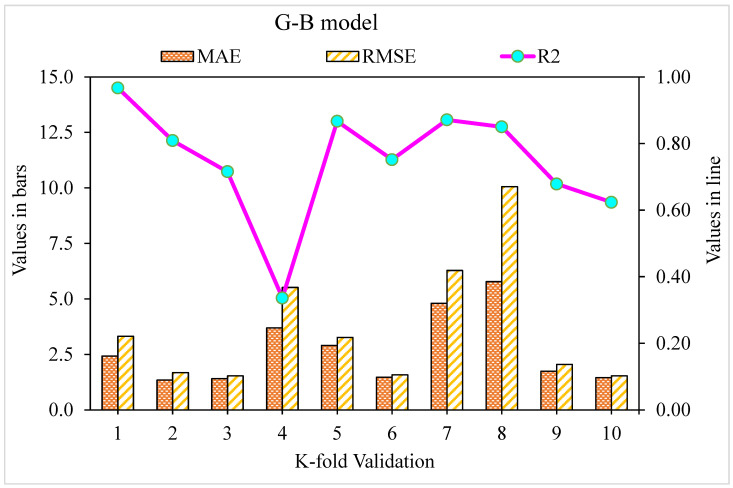
K-fold cross-validation representation for the Gradient Boosting model.

**Figure 11:**
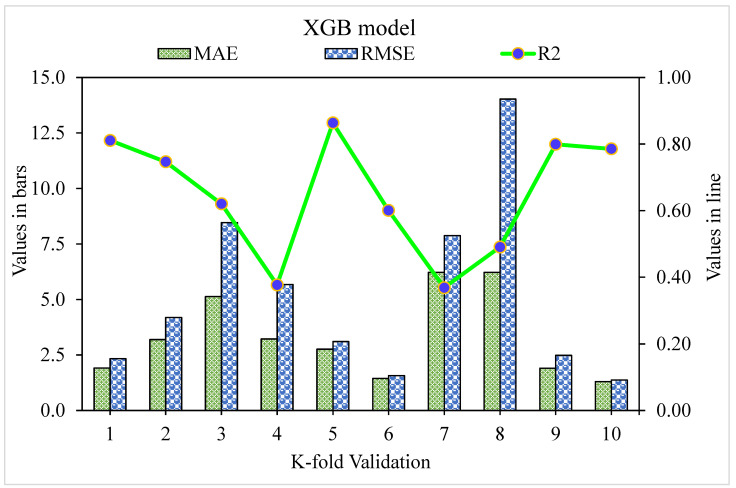
K-fold cross-validation representation for Extreme Gradient Boosting model.

The MAE, RMSE, and R^2^ distributions for the k-fold cross-validation of Random Forest, Gradient Boosting, and Extreme Gradient Boosting models are shown in Figure 9, Figure 10 and Figure 11. The highest, lowest, and average R^2^ values for the R-F model are 0.94, 0.34, and 0.69, respectively, as shown in Figure 9. The maximum, minimum, and average R^2^ values for the G-B model are 0.96, 0.33, and 0.74, respectively, as shown in Figure 10. Figure 11 shows the highest, lowest, and average R^2^ values for the XGB, which were 0.86, 0.36, and 0.64, respectively. The average MAE and RMSE for the R-F model were 2.94 and 4.58, respectively, when the error values were compared. The average MAE and RMSE for the G-B model were 2.7 and 3.68, respectively, whereas the average MAE and RMSE for the XGB model were 3.32 and 5.10, respectively. The G-B model with the lowest error and highest R^2^ value performs the best in forecasting outcomes. Table 3 provides the results of the k-fold study for the models used, including MAE, RMSE, and R^2^ values.

### 4.3. Sensitivity Analysis

**Figure 12:**
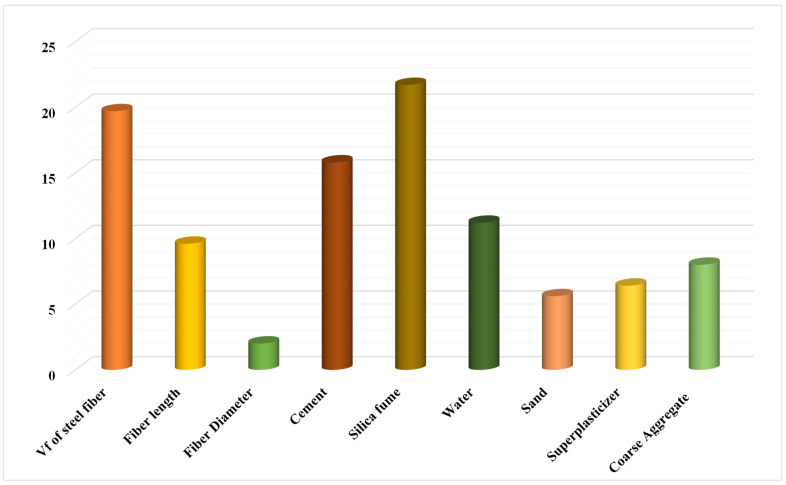
The input variable’s contribution to the forecast.

This research aims to find out how input parameters influence SFRC flexural strength predicting. The influence of input factors on SFRC’s flexural strength prediction can be seen in Figure 12. The essential constituent was determined to be silica fume, which accounted for 21.7% of the total, followed by cement, 15.8%, and superplasticizer, 6.4%. The remaining input components, such as coarse aggregate (8%), water (11.2%), and sand (5.6%), had a lesser influence on the flexural strength of the SFRC forecast. The steel fiber vf, fiber length, and fiber diameter impact were 19.7%, 9.6%, and 2%, respectively. The sensitivity analysis findings were proportionate to the number of input parameters and data points included in the model design. Equations (3) and (4) were used to examine the impact of input variables on model output.

(3) $$N_{i}=f_{max}\left(x_{i}\right)-f_{min}\left(x_{i}\right)$$

(4) $$S_{i}=\frac{N_{i}}{\sum_{j-i}^{n}N_{j}}$$

The highest and lowest projected outputs over the $i^{th}$ output are represented by $f_{max}\left(x_{i}\right)$ and $f_{min}\left(x_{i}\right)$, respectively.

## 5. Discussions

**Figure 13:**
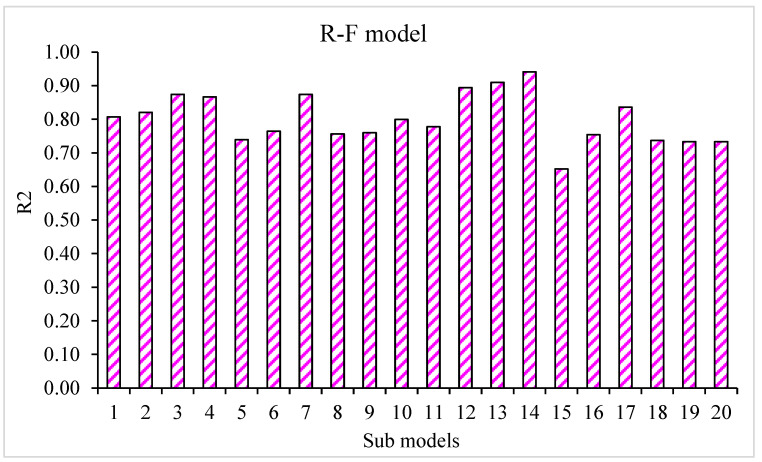
The coefficient correlation (R^2^) values of the R-F sub-model.

**Figure 14:**
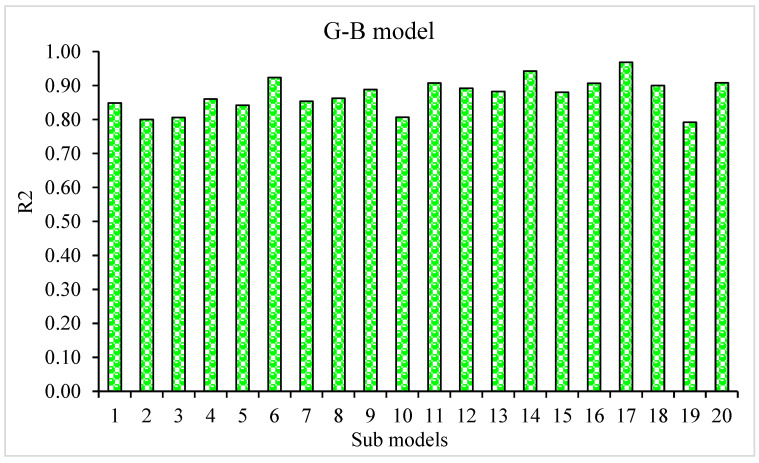
G-B sub-model’s coefficient correlation (R^2^) values.

**Figure 15:**
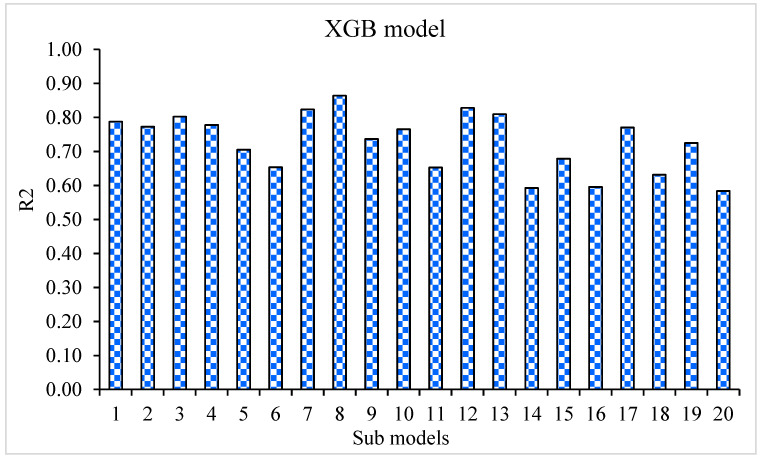
XGB sub-model’s coefficient correlation (R^2^) values.

The goal of this study was to determine how machine learning approaches may be used to forecast SFRC flexural strength. Random Forest, Gradient Boosting, and Extreme Gradient Boosting were three machine learning methods that were investigated. To determine which strategy is the most exact in prediction, the performance of each approach was tested and compared. The G-B model produced a more precise result, with an R^2^ value of 0.96. The R^2^ values of R-F and XGB models were 0.81 and 0.87, respectively. Statistical analysis and the k-fold cross-validation approach were used to confirm the effectiveness of all models. The model works better with minimal error levels. The ML techniques frequently take advantage of the susceptible intern by constructing sub-models, trained on data and maximizing, to boost the value of R^2^. The variation of R^2^ values for sub-models, such as Random Forest, Gradient Boosting, and Extreme Gradient Boosting techniques, is shown in Figure 13, Figure 14 and Figure 15. The Random Forest sub-model had highest, lowest, and mean R^2^ values of 0.94, 0.34, and 0.69, respectively. The Gradient Boosting (G-B) sub-models had maximum, minimum, and average R^2^ values of 0.96, 0.63, and 0.79, respectively. The highest, lowest, and mean R^2^ values for Extreme Gradient Boosting sub-models were 0.87, 0.44, and 0.68, respectively. According to these results, the G-B sub-model is more accurate than the R-F and XGB sub-models. A sensitivity analysis was also performed to evaluate how each input parameter influenced the expected flexural strength of the SFRC. The sensitivity analysis determined how much each of the 10 input variables affects the predicted result.

## 6. Conclusions

This study aimed to put three ensembled ML techniques to the test to estimate the 28-day SFRC flexural strength. Random Forest (R-F), Gradient Boosting (G-B), and Extreme Gradient Boosting (XGB) models were employed to forecast the outcomes. The conclusions of this study are:

- The Extreme Gradient Boosting (XGB) model was less accurate than the Gradient Boosting (G-B) and Random Forest (R-F) models in projecting SFRC flexural strength.
- The Gradient Boosting model outperformed the Extreme Gradient Boosting and Random Forest ensembled machine learning technique in forecasting the 28-days flexural strength of SFRC.
- The Random Forest, Gradient Boosting, and Extreme Gradient Boosting models have a coefficient of determination (R^2^) values of 0.94, 0.96, and 0.86, respectively. All of the models’ outputs are within acceptable bounds, with slight variance from the exact results.
- The k-fold cross-validation test and statistical analysis demonstrated the model’s performance, which revealed that the Gradient Boosting model outperformed the other models investigated in terms of prediction.
- A sensitivity analysis was utilized to determine how much input parameters mattered. It was discovered that Vf of steel fiber, Fiber length, Fiber diameter, Cement, Silica fume, Water, Sand, Superplasticizer, and Coarse Aggregate contributed 19.7%, 9.6%, 2%, 15.8%, 21.7%, 11.2%, 5.2%, 6.4%, and 8%, respectively, to the outcome’s prediction.
- The ensemble machine learning algorithms, especially Gradient Boosting, can effectively estimate concrete strength qualities without requiring long casting and testing.

## Figures and Tables

**Table 1:** Input variables; statistical analysis.

	Mean	Standard Error	Median	Mode	Range	Minimum	Maximum	Count
Cement (kg/m^3^)	451.78	8.37	400	400	509	280	789	173
Water (kg/m^3^)	170.66	2.29	158	152	137	133	270	173
Sand (kg/m^3^)	782.75	11.47	740	835	768	582	1350	173
Coarse Aggregate (kg/m^3^)	927.09	20.63	1050.5	1047	1170	0	1170	173
Superplasticizer (%)	0.91	0.13	0.15	0	5	0	5	173
Silica fume (%)	6.33	0.89	0	0	43	0	43	173
Fly Ash (%)	1.30	0.42	0	0	30	0	30	173
Volume fraction of the hooked steel fiber (%)	0.85	0.05	1	0.5	2	0	2	173
Fiber Length (mm)	40.41	1.21	35	60	60	0	60	173
Fiber diameter (mm)	0.59	0.01	0.615	0.75	0.9	0	0.9	173
Flexural Strength; MPa (28 days)	10.04	0.63	7.82	0	41.7	0	41.7	173

**Table 2:** Statistical analysis of the approaches used.

Models	MAE (MPa)	RMSE (MPa)	R^2^
Random Forest	1.5	2.0	0.94
Gradient Boosting	1.3	1.8	0.96
XGBoost	2.4	3.3	0.86

**Table 3:** K-fold cross-validation results.

K-Fold	Random Forest			Gradient Boosting			Extreme Gradient Boosting		
	MAE	RMSE	R^2^	MAE	RMSE	R^2^	MAE	RMSE	R^2^
1	2.10	3.74	0.90	2.43	3.32	0.97	1.91	2.33	0.81
2	3.45	4.66	0.95	1.35	1.68	0.81	3.20	4.19	0.75
3	2.75	4.03	0.75	1.40	1.53	0.72	5.14	8.47	0.62
4	3.72	5.90	0.36	3.69	5.51	0.34	3.22	5.67	0.38
5	4.74	7.12	0.86	2.91	3.26	0.87	2.76	3.10	0.86
6	1.29	1.65	0.74	1.47	1.58	0.75	1.44	1.57	0.60
7	1.92	2.04	0.35	4.80	6.28	0.87	6.21	7.88	0.37
8	6.65	13.02	0.79	5.78	10.05	0.85	6.22	14.03	0.49
9	1.24	1.79	0.39	1.74	2.05	0.68	1.90	2.49	0.80
10	1.54	1.87	0.88	1.45	1.53	0.62	1.30	1.37	0.79

## Data Availability

Data will be made available upon request from corresponding author.
